# Does hemispheric vascular regulation differ significantly in glaucoma patients with altitudinal visual field asymmetry? A single-center, prospective study

**DOI:** 10.1007/s10792-021-01876-0

**Published:** 2021-05-19

**Authors:** David Kuerten, Konstantin Kotliar, Matthias Fuest, Peter Walter, Muriel Hollstein, Niklas Plange

**Affiliations:** 1 Department of Ophthalmology, Uniklinik RWTH Aachen, Pauwelsstr. 30, 52074 Aachen, Germany; 2 Department of Medical Engineering and Technomathematics, FH Aachen, Aachen, Germany

**Keywords:** Glaucoma, Visual field asymmetry, Ocular blood flow, RVA, Vascular response, Neurovascular coupling

## Abstract

**Purpose:**

Vascular risk factors and ocular perfusion are heatedly discussed in the pathogenesis of glaucoma. The retinal vessel analyzer (RVA, IMEDOS Systems, Germany) allows noninvasive measurement of retinal vessel regulation. Significant differences especially in the veins between healthy subjects and patients suffering from glaucoma were previously reported. In this pilot-study we investigated if localized vascular regulation is altered in glaucoma patients with altitudinal visual field defect asymmetry.

**Methods:**

15 eyes of 12 glaucoma patients with advanced altitudinal visual field defect asymmetry were included. The mean defect was calculated for each hemisphere separately (−20.99 ± 10.49 profound hemispheric visual field defect vs −7.36 ± 3.97 dB less profound hemisphere). After pupil dilation, RVA measurements of retinal arteries and veins were conducted using the standard protocol. The superior and inferior retinal vessel reactivity were measured consecutively in each eye.

**Results:**

Significant differences were recorded in venous vessel constriction after flicker light stimulation and overall amplitude of the reaction (*p* < 0.04 and *p* < 0.02 respectively) in-between the hemispheres. Vessel reaction was higher in the hemisphere corresponding to the more advanced visual field defect. Arterial diameters reacted similarly, failing to reach statistical significance.

**Conclusion:**

Localized retinal vessel regulation is significantly altered in glaucoma patients with asymmetric altitudinal visual field defects. Veins supplying the hemisphere concordant to a less profound visual field defect show diminished diameter changes. Vascular dysregulation might be particularly important in early glaucoma stages prior to a significant visual field defect.

## Introduction

Glaucoma is an optic disc neuropathy, characterized by optic disc cupping, loss of vital nerve fibers and visual field defects. Although being one of the leading causes of blindness the exact mechanisms and pathophysiology of glaucoma is still not understood today [1]. Often heatedly discussed, but widely accepted, ocular hemodynamics and ocular blood flow appear to be a critical factor in the pathogenesis of glaucoma [2–4]. An ischemia–reperfusion damage vicious cycle has been proposed and also disturbances in the venous part of the circulation have regularly been found in glaucoma patients [5–8]. Changes in ocular blood flow (OBF) can precede visual field loss and may play a major role during the earliest stages of glaucoma [9]. Differences in OBF were reported between early glaucoma patients and healthy subjects, whereas the changes in middle- to late-stage glaucoma were minor [9]. Lately, automated assessment of retinal vessel diameters showed significant alterations in glaucoma [10–13]

Furthermore, retinal vessel narrowing has been correlated with glaucoma progression in large population-based studies [7–9].

Dynamic Measuring and Assessing ocular blood flow remain a challenge to date. Often surrogates for ocular blood flow are used [14].

Vascular dysregulation refers to the regularly recorded inadequate vascular responses in glaucoma patients [6, 8]. Vessel diameter is regulated by a complex system with many contributors (e.g. autonomic nervous system). Retinal vessel narrowing, found in the primary vasospastic syndrome, may affect visual field defects [5]. The finding resulted in the assumption, shared by many experts, that vascular dysregulation and particularly vasospasms play a role in glaucoma [15].

The Retinal Vessel Analyzer (RVA) allows for continuous measurement of retinal vessel diameters. An innovation in the currently available system is the dynamic vessel analysis (DVA). The characteristic response of the retinal vessels to flicker light stimulation allows assessment of vascular autoregulation and neurovascular coupling. Neurovascular coupling describes the instant adaptation of blood supply to the increased activity-level of neural tissue. With increased neural activity, Neurotransmitters are released by the activated neurons, initiating a complex sequence leading to a significant raise of blood supply for the neural tissue. Neurons, astrocytes and endothelial cells mediate this response as a functional unit and malfunctions in this functional circle might be a contributor in the vascular dysfunction suspected in glaucomatous eyes. The RVA system was highlighted in many studies as a potent tool in vascular research [16]. Early vascular changes in diabetes as well as arterial hypertension and other vascular disorders can be recorded with this noninvasive technique [17, 18]. Studies showed that vessel diameter and vascular regulation are impaired in open angle glaucoma [8, 19].

The previous studies, using the device, focused on “global” alterations, whereas it might be particularly interesting, if localized differences can be recorded. This might help our understanding of the complicated interaction of blood flow regulation and glaucomatous damage.

In this pilot study we wanted to investigate if intraocular differences in vascular autoregulation can be recorded in patients with asymmetric hemispheric visual field defects in-between the more affected and less affected hemisphere.

## Subjects and methods

### Patients

Following the tenets of the Declaration of Helsinki, every study subject gave their informed consent to participate in this study and the study approved by the local ethic committee of the University Hospital RWTH Aachen (EK 293/16).

A detailed medical and ophthalmic history was recorded for all participants.

All participants received a complete ophthalmological examination prior to all measurements, consisting amongst others of intraocular pressure measurements with Goldmann applanation tonometry, achromatic visual field defect testing (Humphrey visual field analyzer, Zeiss, Germany) and peripapillary retinal nerve fiber layer (RNFL) thickness via spectral domain optical coherence tomography (SD-OCT, Heidelberg Engineering, Heidelberg, Germany) measurement. All visual field examinations with the achromatic standard program of the Humphrey Visual Field Examiner (Zeiss, Germany) were performed on the same day prior to any RVA measurements. The mean deviation (MD) was calculated for each hemisphere for analysis. For retinal nerve fiber thickness, the nasal and temporal superior and inferior sectors were aggregated and an average for superior and inferior retinal nerve fiber thickness was calculated. Cup/Disc—Ratio was determined clinically by the same experienced investigator (NP).

The exclusion criteria are as follows: higher degrees of cataract or corneal diseases with a profound clouding of the cornea and severe vitreous opacity limiting the imaging of retinal vessels. Furthermore, patients with a history of epilepsy, angle closure glaucoma or shallow anterior chamber patients with a risk of excessive IOP increase after mydriasis, inability to perform the examinations, uncontrolled arterial hypertension, presence of any other severe ocular diseases apart from glaucoma, high ametropia (spherical equivalent < − 5 dpt or >  + 3 dpt), high astigmatism (>2 dpt), visual field defects due to other causes apart from glaucoma (e.g. cerebral apoplexia, anterior ischemic optic neuropathy) were excluded.

None of the patients were asked to cease any systemic medication that might affect the vascular reaction prior to the measurement as paired intraocular differences were measured and any side effects of systemic medication were supposed to influence both hemispheres similarly.

A glaucomatous optic disc cupping (precisely: thinning of the inferior and/ or superior rim, cup-to-disc ratio asymmetry of >0.2 not due to optic disc size asymmetry) in combination with compatible glaucomatous visual field deficits (in detail: a cluster of three or more test points with >5 dB or two points with >10 dB sensitivity reduction compared to age-eccentricity-corrected normal value) formed the base for the diagnosis of POAG (European Glaucoma Society 2008).

The altitudinal visual field defect asymmetry was defined as a difference of >10 dB in mean defect (MD) in-between the hemispheres. The mean deviation of the affected hemisphere was −20.99 ± 10.49 db, whereas the unaffected hemisphere showed a mean deviation of −7.36 ± 3.97 (paired *t*-test *p* < 0.001).

Overall value of the mean defect (MD) was not a selection criteria. 15 eyes of 12 patients (mean age 69.5 ± 8.1 years) suffering from primary open angle (POAG) and pseudoexfoliation (PEX) glaucoma with asymmetric visual field defects were included in this pilot prospective clinical pilot study.

The topical antiglaucomatous medication consisted of (dorzolamide, latanoprost, timolole, brimonidine and combinations thereof).

### Retinal vessel analyzer

In preparation for RVA measurements adequate pupil dilation was achieved with 2 to 3 eyedrops of mydriaticum (Pharma Stulln, Germany) 30 min prior to any measurements.

Blood pressure and heart rate values were recorded prior and after each measurement with the patient sitting upright and resting for 5 min prior to any measurements.

The RVA, version DVAlight (dynamic vessel analyzer, IMEDOS Systems, Jena, Germany) is a commercially available unit, consisting of a fundus camera (Zeiss, Germany), video capturing and recording device, a real-time monitor connected to a personal computer with video processing software. The RVA and its technical features are presented in detail elsewhere [16, 20]. In short, vessel diameters of the retinal arterioles and venules are measured longitudinally at an observer-defined location of the fundus. The device relies on the measuring principle that inside retinal blood vessels is a column of red blood cells, which can absorb one part of the light and the RVA measures the diameter of the column of these red blood cells. The integration of a video recording software into the device allows offline reassessment of the recorded data. The retinal vein and artery diameters are recorded precisely with a temporal resolution of 25 readings per second. The retinal vessel diameter is measured continuously. The predefined segments are chosen in the main vessel branches approximately one disc diameter away from the rim of the optic disc (ONH), to minimize disturbances due to blood flow turbulences. The patient is sitting upright with the head in a headrest and asked to look slightly up or down to a fixation needle depending on which vessels are captured. The fundus is illuminated with green light of a wavelength between 567 and 587 nm. Flicker stimulus is generated by interrupting the illuminating light with a optoelectric shutter at a frequency of 12.5 Hz, which is in the range of the optimal excitation frequency for retinal vessels in humans. [16] The flicker stimulation relies on the principle of neurovascular coupling [21, 22].

The device produces measurements with a high repeatability and reproducibility [16].

The standard measurement protocol consists of 3 episodes with 20 s flicker stimuli followed by 80 s of observation and took overall 350 s.

The vessels in the retinas’ superior half 1–2 ONH- diameters from the ONH-rim were measured first and after a pause of 30 min the vessels in the lower half were measured. In previous studies leading up to this work no significant differences were recorded, when measuring either the upper or the lower hemisphere first in healthy test subjects (unpublished data).

For the statistical analysis, the hemisphere of the visual field was assigned to the corresponding retinal vessel reaction.

### Data evaluation

The data were analyzed separately with a template with corresponding macros in a spreadsheet (MS Excel, Microsoft, USA). With the template, each numerical data provided from the RVA was filtered, processed and analyzed. For statistical purposes, absolute vessel diameters of the predetermined segments were calculated individually during the last 30 s before the first flickering [16]. It was measured in MU, where 1MU corresponds to 1 µm, in the Gullstrand’s eye. The three single curves for each individual consisting of 30 s baseline assessment prior to flickering, 20 s of flicker application and 80 s thereafter were averaged. The averaged response temporal vessel response for each individual was calculated as a mean of these 3 curves. The absolute diameter recorded in the 30 s before the first flicker was set to 100%, and all changes were recorded in % to the individual baseline value. For each individual, this normalized averaged time course of relative vessel diameter changes was smoothed using the running median (5 s frame) and the corresponding back shift. The following parameters of dynamic retinal vessel reaction were derived for each subject:

1. Mean maximal dilatation in response to flicker stimulation (absolute maximum of the smoothed normalized curve, (in % to the baseline)
2. Time until maximal vessel dilatation (in s)
3. Mean maximal constriction after flicker stimulation (absolute minimum of the smoothed normalized curve) For the curves under the 100%-line the value was negative; (in % to the baseline)
4. Time until maximal vessel constriction (in s)
5. Amplitude of the reaction (in % to baseline calculated as the difference between mean maximal dilation and constriction on the smoothed curve)

In short, the reaction patterns of the retinal arteries and veins show a bimodal reaction with 2 dilatation peaks during flicker light stimulation followed by a constriction after the flicker stimulation before returning to baseline diameter.

### Statistical analysis

Statistical Analysis and graph creation were performed with MS Excel (2000 for Windows) and Graph Pad Prism (Software 7.0 for Windows) and *p* < 0.05 was defined as statistically significant and *p* < 0.1 as a trend. Due to the non-normally distributed data, differences between the hemispheres were analyzed using a Wilcoxon Rank test. Descriptive statistics of non-normally distributed data are given by median and interquartile range (IQR = 1st to 3rd quartile). Because of the explorative character of this pilot study no correction for multiple comparisons was performed.

## Results

7 of the 12 patients were pseudophakic. Five were phakic with not severe stages of cataract. Three patients had undergone glaucoma surgery performed 1 to 5 years prior to the measurements. One patient had undergone laser–trabeculoplasty 2 years prior to our measurements.

7 of the 12 patients suffered from treated arterial hypertension. The regularly taken medication consisted of ace—inhibitors (6/7 patients), calcium—antagonists (3/7) and beta—blockers (2/7). Two patients suffered from diabetes. One of the two patients had to take insulin daily. No smokers where among the 12 patients. One patient reported a history of vasospasms affecting the hands, whereas one other patient had suffered from migraine in the past. High blood fats were known in two patients, both took simvastatine.

No significant differences were recorded in blood pressure values prior to and after the RVA measurements (*p* > 0.2).

Table 1 shows the visual field defect mean deviation (MD) in the more affected and less affected hemisphere. In 13 eyes included in this pilot study the profound visual field defect was found in the upper hemisphere. The affected hemisphere showed a significantly higher visual field MD (*p* < 0.001). The overall MD was -13.41 ± 3.76 dB.

**Table 1:** The basic data of all eyes (including visual field mean defect and RNFL)

Visual field defect: MD (in dB)		Average nerve-fiber thickness in nasal- and temporal-inferior/superior sectors (in µm)		IOP (in mmHg)	Cup-to-disc-ratio
No defect	HS defect	No defect	HS defect	15.7 ± 4.0	0.81 ± 0.13
−4.8 (−9.19– − 1.27)	− 23.73 (− 27.88– − 17.73)	71.5 (66.5–86.5)	46.5 (42–54)		
**WRT p < 0.001**		**WRT p < 0.001**			

Data provided either as median (1st–3rd quartile), or medial defect ± standard deviation

*WRT* Wilcoxon rank test, *HS* Hemisphere, *MD* Mean deviation, *IOP* intraocular pressure

The average nerve fiber-layer thickness in the superior / inferior sectors was significantly lower corresponding to the localization of the profound visual field defect (*p* < 0.001). The average IOP and Cup-to-disc–ratio values are presented as well.

**Fig. 1:**
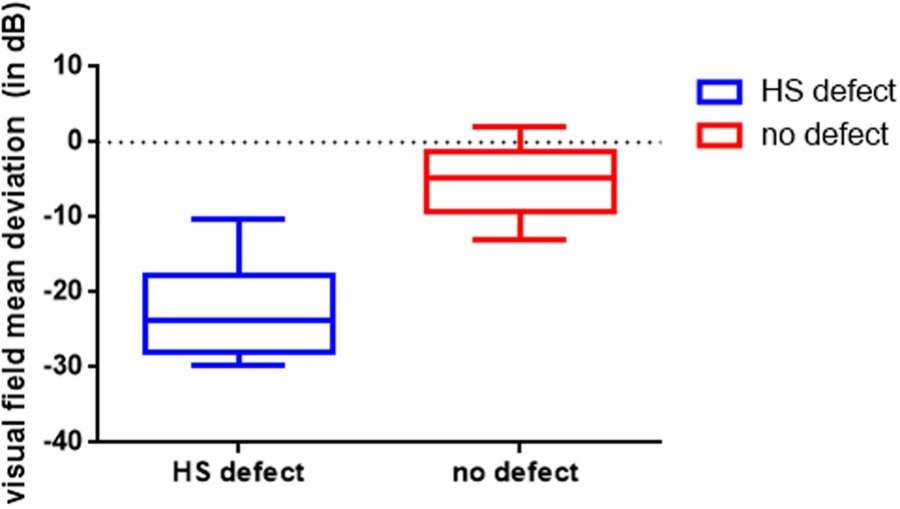
Is showing the box plot for mean visual field defect of either the affected or unaffected hemisphere

**Fig. 2:**
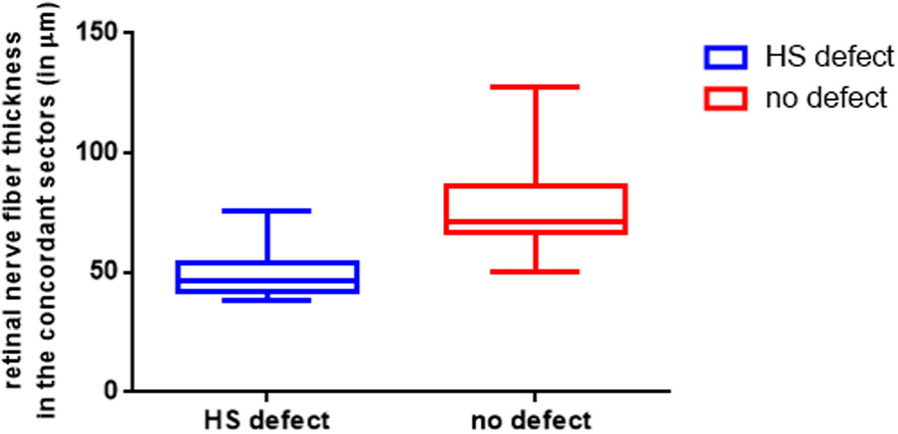
Is showing the average retinal nerve fiber thickness in the concordant superior/inferior sectors (recorded via SD–OCT)

Mean ± Standard Deviation values for hemispheric MD and RNFL-thickness are shown in Fig. 1 and 2.

Table 2 shows the venous and Table 3 the arterial RVA-results.

**Table 2:** Provides the RVA parameters of the venous reaction to flicker light and the absolute vessel diameter

Mean maximal dilatation (in % to baseline)		Time until maximal dilatation (in s)		Mean maximal constriction (in % to baseline)		Time until maximal constriction (in s)		Amplitude of the reaction (in % to baseline)		Absolute vessel diameter (in MU)	
No defect	HS defect	No defect	HS defect	No defect	HS defect	No defect	HS defect	No defect	HS defect	No defect	HS defect
5.1 (3.7–8.2)	6.6 (6.2–8.5)	19 (14–23)	19 (13–23)	− 0.5 (− 1.5–0)	− 1.7 (− 3.7– − 0.8)	75 (52–86)	60 (47–87)	6.7 (3.5–8.4)	8.4 (5.9–12.9)	124.8 (120.1–144.7)	121.6 (116.7–129.6)
WRT *p* = 0.05		WRT *p* = 0.31		**WRT p = 0.04**		WRT *p* = 0.21		**WRT p = 0.02**		WRT *p* = 0.42	

Mean (1st quartile–3rd quartile)

Significant correlations are in bold print

*WRT* Wilcoxon rank test, *MU* Measurement units, *HS* Hemisphere

**Table 3:** provides the RVA parameters of the arterial reaction to flicker light and the absolute vessel diameter

Mean maximal dilatation (in % to baseline)		Time until maximal dilatation (in s)		Mean maximal constriction (in % to baseline)		Time until maximal constriction (in s)		Amplitude of the reaction (in % to baseline)		Absolute vessel diameter (in MU)	
No defect	HS defect	No defect	HS defect	No defect	HS defect	No defect	HS Defect	No defect	HS defect	No defect	HS defect
3.3 (1.6–4.6)	5.3 (3.3–7.8)	15 (12–23)	20 –(17–22)	− 2.2 (− 5– − 0.8)	− 3 (− 4.3– − 2.3)	46 (37–59)	47 (34–67)	2.2 (0.03–3.6	3.9 (2.4–5.9)	108.6 (95.9–110.8)	100 (91.7–107.4)
WRT *p* = 0.09		WRT *p* = 0.33		WRT *p* = 0.42		WRT *p* = 0.92		WRT *p* = 0.16		WRT *p* = 0.35	

Mean (1st quartile–3rd quartile)

Significant correlations are in bold print

*WRT* Wilcoxon rank test, *HS* Hemispheric, *MU* measurement units

Vessel diameters did not differ significantly in-between the upper and lower (i.e. more or less affected) hemisphere (please refer to Table 2 and 3).

Using the Wilcoxon Rank Test significant differences in-between the hemispheres in each patients’ eye were revealed for several RVA-parameters.

The amplitude of dilatation was significantly higher in the veins of the hemisphere corresponding to the more severe visual field defect 8.4 (5.9–12.9) vs (6.7 (3.5–8.4), *p* = 0.02). Venous constriction after flicker light stimulation was significantly stronger in the more-affected hemisphere -1.7% (−3.7 to −0.8) vs. (−0.5% (−1.5 to 0), *p* < 0.04). Mean maximal venous dilation was higher in the more-affected hemisphere 6.6% (6.2–8.5) vs. (5.1% (3.7–8.2), *p* = 0.05).

The retinal arteries seem to show similar vessel reactions to flicker light. None of the parameters reached statistical significance (Table 3).

**Fig. 3:**
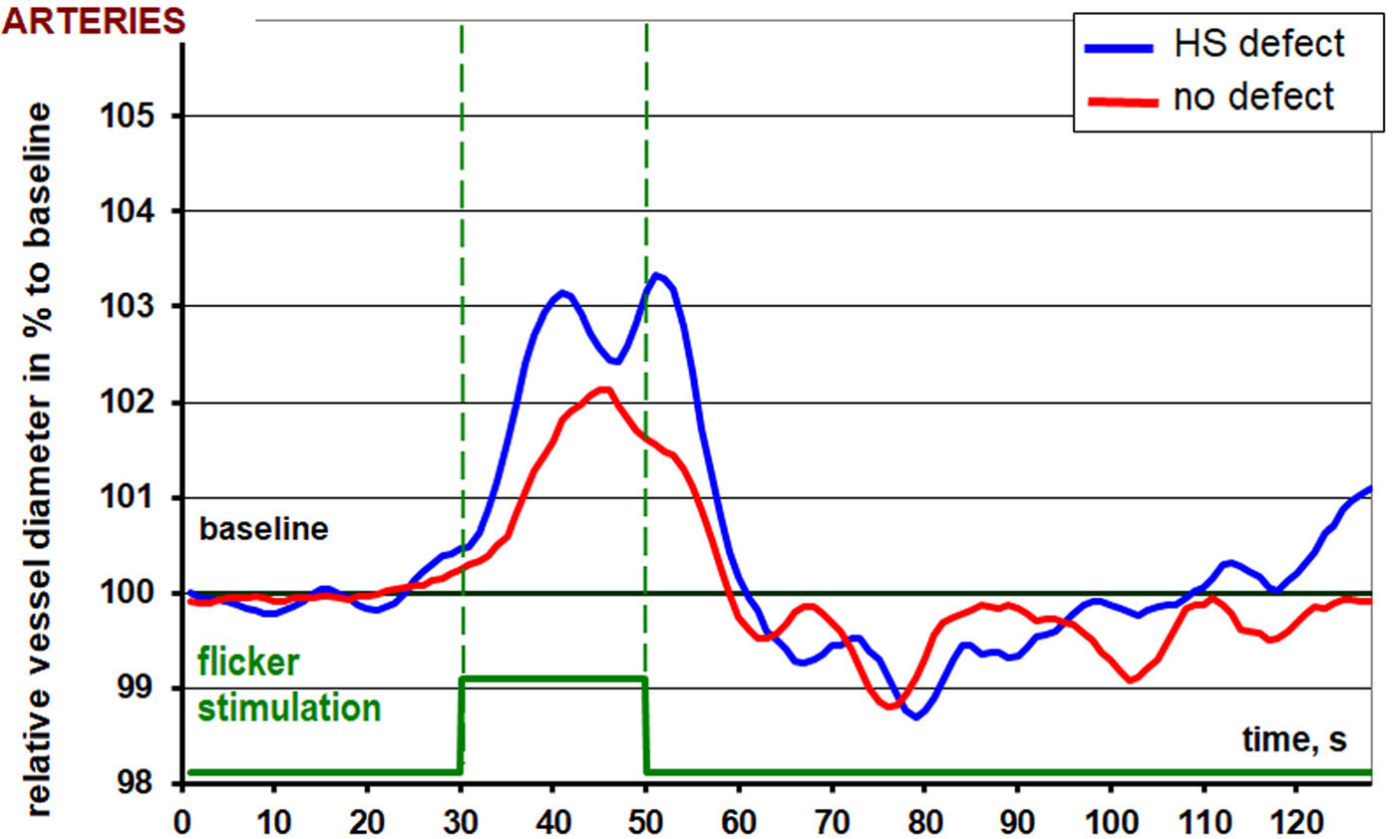
Is showing the RVA-results for the arteries of both hemispheres. HS defect is the graph for the arteries concordant to the severe altitudinal visual field defect. No defect is the graph for the arteries concordant to the unaffected hemisphere

**Fig. 4:**
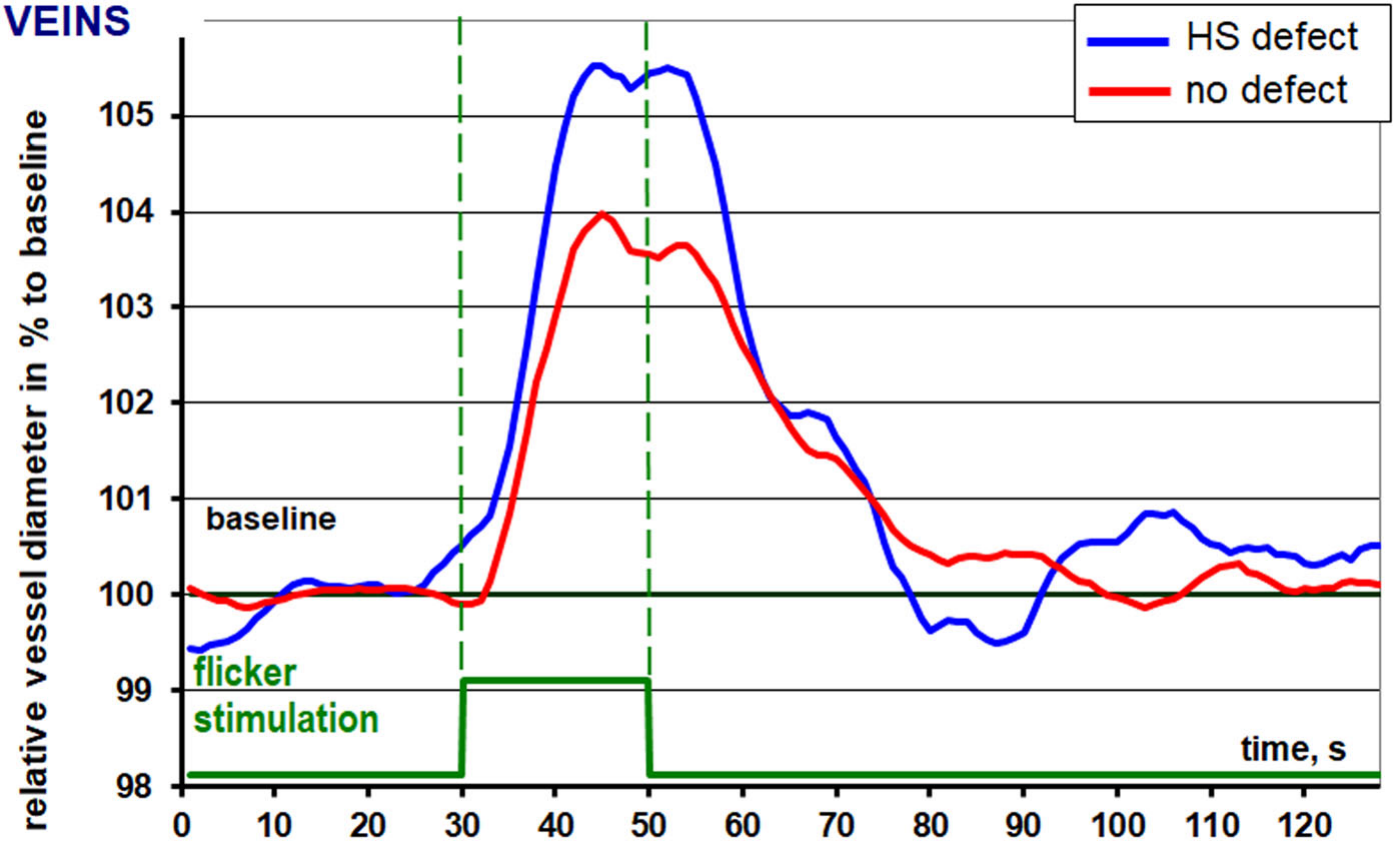
Is showing the RVA-results for the veins of both hemispheres. HS defect is the graph for the veins concordant to the severe altitudinal visual field defect. No defect is the graph for the veins concordant to the unaffected hemisphere

The overall configuration of the arterial reaction to flicker light stimulation recorded differs significantly in-between the hemispheres in this study. Ordinarily a bimodal response can be recorded with two dilatation peaks in close proximity and a consecutive decrease in vessel diameter leading to a vessel constriction before returning to the baseline diameter. However, the highest dilatation in the less-affected hemisphere is achieved earlier almost exactly in between the “normal” bimodal response in the hemisphere concordant to the severe visual field defect (Fig. 3). No altered configuration was recorded for the veins in response to flicker light in patients in this pilot study, however maximal dilatation and constriction are lower in vessels concordant to the less severe hemispheric visual field defect (Fig. 4).

## Discussion

In our study we were able to show, that intraocular differences in retinal vascular regulation can be recorded in glaucoma patients with profound altitudinal visual field defect asymmetry. Interestingly, the venous flicker response was lower in the hemisphere compliant with a less severe visual field defect in terms of overall amplitude of vessel reaction (6.7 vs 8.4%) as well as constriction after flicker stimulation (−0.5 vs. −1.7%). Arterial diameter changes failed to reach statistical significance but reacted in a similar manner.

Being counterintuitive at first (higher amplitude of vessel reaction in hemispheres concordant to a severe altitudinal visual field defect), our findings support the previous reports about impaired OBF particularly in early glaucoma stages and minor to no changes in advanced stages [6]. The hemisphere compliant to a less severe visual field defect i.e. an early glaucoma stage showed lower dilatation as well as constriction in the venous system. The exact mechanisms on why vascular dysregulation is particularly altered in the less affected hemisphere (i.e. an early glaucoma stage) remains not completely understood today. Garhofer et al. showed in 2003[23], that high doses of lactate reduce the flicker response, whereas low doses increase it. It is likely that lower concentrations of lactate arise in the hemisphere with less retinal activity i.e. the hemisphere compliant to a profound altitudinal visual field defect and thereby higher flicker responses can be recorded. Furthermore, anatomical changes in the astrocytes have been found in animal models of glaucoma [24]. In late stages of glaucoma the astrocytes were thickened, showed simplified processes and reduced spatial coverage, whereas localized sprouting of new processes was recorded in early stages of the disease, before detectable changes in ganglion cell numbers. Astrocytes are important in the flicker light induced vascular response. Therefore, changes in the astrocytes in-between the hemispheres might contribute to the detectable locally different reaction.

Furthermore, venous constriction was significantly higher in the hemisphere corresponding to a significant visual field defect, which may be the main reason for the significant differences in amplitude of the venous reaction. A higher venous constriction can be sign of vascular dysregulation in this hemisphere, therefore we believe that vascular regulation does not “normalize” in further progressed stages of glaucoma.

Finally, it is known that vascular diameter decreases in further progressed glaucoma stages. No significant changes were found in our study; however, the small sample size should be taken into account. Thinner baseline vessels should have a higher “reserve” for dilatation. Thereby higher dilatation and constriction recorded in our patients, might be a result of thinner baseline diameters to begin with.

Nevertheless, we were able to record similar results in glaucoma patients with intraocular differences, whereas the more progressed eye showed higher responses to flicker light stimulation, while also no significant differences in vessel diameter were recorded (unpublished data).

Like our findings of significant differences in venous reaction, reduced venous responses to flicker light stimulation in early glaucoma stages have been published in the past.

Garhofer et al. [8] showed in their study on 31 patients with early stages of open angle glaucoma that especially flicker induced retinal venous dilatation is greatly diminished in glaucoma patients compared to healthy controls. However, the study was unable to find significant differences in the arterial vascular response. The findings are not completely comparable to our results as the parameter of flicker stimulation were different.

Nagel et al. [25] found significantly impaired retinal venous dilatation after artificial IOP rise in POAG patients in comparison to healthy controls and ocular hypertension patients. The authors interpreted the findings similarly as a sign of disturbed vascular regulation in glaucoma.

Gugleta et al. [19] reported that the retinal vessel response to flicker light is impaired in glaucoma patients. Forty-seven patients suffering from POAG were compared to 46 OHT patients and 56 age-matched healthy controls. The responses of both arteries as well as veins were significantly lower in POAG patients compared to both other groups. No significant differences in-between both POAG eyes were recorded. However, both eyes had an early visual field defect in that study (MD 3.4 ± 3.2 for the “worse” eye and 1.4 ± 2.1 dB in the other eye). The level of glaucomatous damage was not significantly correlated to vascular responses recorded with the RVA.

An important confounding factor in many studies investigating ocular blood flow and glaucoma is the application of topical antiglaucomatous medication, as no topical medication is taken by the healthy controls. One should consider that the exact influence of topical antiglaucomatous medication on ocular blood flow and vascular responses is not completely understood. It appears likely, that e.g. topical beta-blockers do impair the vascular regulation and response in RVA measurements. Furthermore, matching for systemic diseases and systemic medication, that might influence RVA measurements can be challenging.

These limitations make the investigation of intraocular differences more rewarding, as the influence of confounding factors can be neglected in a paired intraocular comparison. Therefore, systemic vascular disease, vascular dysregulation, systemic medical treatment or antiglaucomatous medication will probably affect both retinal hemispheres of the patient with glaucoma in a similar way.

The question arises, if light-induced alterations in vascular regulation recorded with the RVA in glaucomatous eyes, can be conferred to general vascular dysregulation besides the experimental setting. It is not known to date if pressure-related vascular regulation and the experimental light-induced vascular regulation are using exactly the same pathways and mechanisms. It appears likely that they do, or rather that disturbances recorded in the experimental setting can have a real-life implication for the patient, but clear evidence has not been provided to date. Waldmann et al. reported in 2016, that if the vessel reaction (in arteries as well as veins) to a flickering stimulus in one hemisphere was weaker than in the opposite one, it would indicate a higher rate of RNFL thinning over time [6]. These results also support our observation, that retinal blood flow regulation can be altered altitudinally and that localized dysregulation can be recorded in glaucomatous eyes.

Previously, Arend et al. [26] reported significantly prolonged arterial-venous passage times recorded via fluorescein angiography in NTG patients with altitudinal asymmetric visual field defects, also highlighting localized blood flow alteration in glaucoma eyes. The authors concluded that circulatory deficits of the retinal tissue are linked to the visual field loss and the improvement of these circulatory deficiencies might be beneficial for treatment of glaucoma. In fact, one has to consider the significant difference between reduced blood flow and reduced vessel reactivity as a sign of vascular dysregulation.

Furthermore, we have reported that significantly different ocular blood flow parameters recorded via color-Doppler-Imaging can be recorded in POAG patients with significant interocular differences in visual field defect [27].

To date it is not completely understood if these reported inter-/intraocular differences can be attributed solely to diminished retinal function and thereby reduced metabolic needs and reduced neurovascular coupling in eyes / hemispheres with higher degrees of visual field loss. It is likely, that disturbed ocular blood flow and vascular dysregulation might be underlying factors causing the more profound visual field defects and disease progression as highlighted by the published findings e.g. of Waldmann et al. [6].

The disturbed configuration of the arterial flicker response in the less affected hemissphere has not been reported to date. We believe that this finding is another sign of vascular dysfunction in glaucoma. Further studies are necessary to investigate the changes of flicker reaction, i.e. neurovascular coupling in progressive diseases over time.

When comparing RVA findings one should consider that an age dependent decrease in the myogenic response of retinal arterioles is known. It was first described by Jeppesen et al. [28] and Nagel et al. [29] reported similar findings. Overall, the 12 patients in our study were of similar age. Nevertheless, an age-related reaction impairment in the vascular reaction patterns in our patients cannot be ruled out fully. Therefore, larger scaled studies are in the works to answer these questions in greater detail.

One limitation in our study, beside the limited number of subjects and its’ implication on the statistical validity, is that different glaucoma entities were included in our study population. One patient suffered from PEX-glaucoma and 11 patients from POAG. Although similar blood flow impairments were reported via CDI for these glaucoma entities [30], it is still not completely clear if the same can be said about different vascular reaction patterns via RVA. We found no significant differences in the RVA parameters measured in the upper and lower hemispheres in healthy patients previously (unpublished data). However, it is not known, if the same can be said about glaucoma eyes without asymmetric visual field defects.

Furthermore, we performed an explorative pilot study, the power of the study was not enough to show significant differences for arteries, but an effect of visual field asymmetry on venous flicker reaction was recorded.

Overall, vascular reaction to flicker light does not seem to be diminished in direct relation with the extent of the visual field defect. The venous vessels of the more affected hemisphere showed a higher flicker response, than the less affected hemisphere. This observation might be one of the reasons, why no study has been able to correlate RVA recordings to the level of glaucomatous damage to date. Vascular dysregulation might be particularly important early on and glaucomatous eyes and “normalizes” further on in the progression of the disease. Our findings highlight, how poorly vascular dysregulation in glaucoma is understood today.

One insight provided by our research is, that it appears to be important to report the localization of visual field defects in glaucoma patients in RVA studies. As we have shown in this pilot study, hemispheric visual field defects lead to altered vascular reaction towards flicker light. Therefore, measuring the same hemisphere in all glaucoma patients independent of the visual field defects’ localization might be impairing the results in other RVA studies.
